# Congenital absence of the left circumflex coronary artery and an unusually dominant course of the right coronary artery

**Published:** 2010

**Authors:** MURAT BASKURT, BARIS OKCUN,, ILKER M CAGLAR, ALEV A OZKAN, MURAT ERSANLI, TEVFİK GURMEN

**Affiliations:** Cardiology Department, Institute of Cardiology, Istanbul University, Istanbul; Cardiology Department, Institute of Cardiology, Istanbul University, Istanbul; Cardiology Department, Institute of Cardiology, Istanbul University, Istanbul; Cardiology Department, Institute of Cardiology, Istanbul University, Istanbul; Cardiology Department, Institute of Cardiology, Istanbul University, Istanbul; Cardiology Department, Institute of Cardiology, Istanbul University, Istanbul

**Keywords:** angiography, coronary heart disease, coronary vessels

## Abstract

Congenital absence of the left circumflex artery (LCX) is a very rare congenital anomaly of the coronary circulation, and only a few cases have been reported in the literature. We report on a 55-year-old female with atypical chest pain. Routine coronary angiography showed a normal left anterior descending coronary artery (LAD), no LCX and a dominant right coronary artery (RCA), which continued beyond thecrux, running the full course of the LCX and terminating in the left atrial branch. Neither aortography nor pulmonary angiography showed a separate ostium for the LCX. There were no atherosclerotic lesions in the coronary arteries, or ischaemia on stress myocardial perfusion imaging. Multidetector row computed tomography (MDCT) was performed to confirm the diagnosis.

## Introduction

Various coronary artery anomalies have been described in the literature, with a range of occurrence from 0.6 to 1.3%, and mostly diagnosed incidentally.1 Congenital absence of the left circumflex coronary artery (LCX) is an extremely rare condition, and only a few cases have been reported.^2^-^16^ We report on a patient with atypical symptoms, an absent LCX and superdominant right coronary artery (RCA).

## Case Report

A 55-year-old female with atypical chest pain for the previous two years was admitted to our hospital. She had had type 2 diabetes for 10 years. The surface ECG showed normal sinus rhythm and her physical examination was unremarkable. Chest X-ray and two-dimensional echocardiography were normal.

**Fig. 1.**
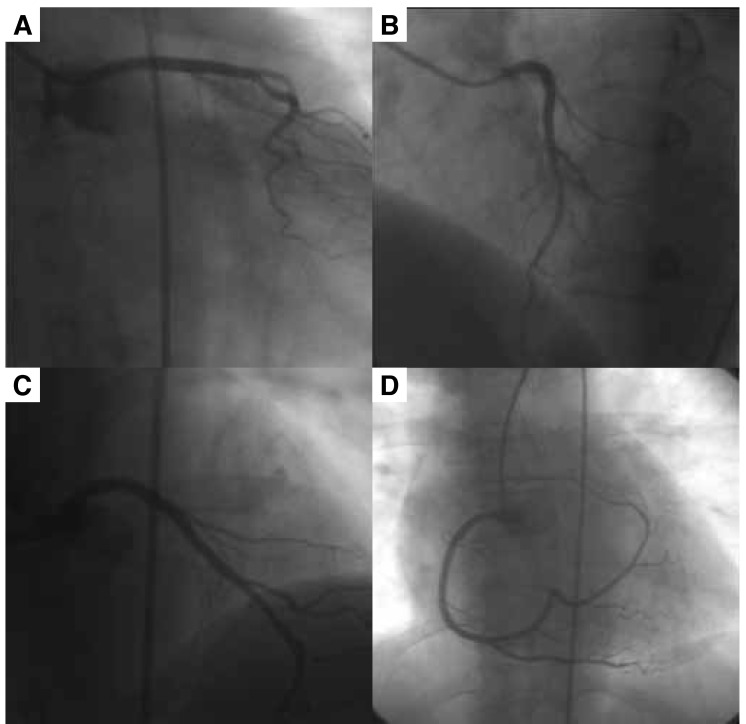
The left circumflex artery is not seen in various views in conventional coronary arteriography. (A) The left circumflex artery is absent in this view (LAO 0ø, caudal 20ø). Note that the contrast in the left sinus of Valsalva does not indicate another possible ostium of the circumflex artery. (B) In this view the left anterior descending artery is clearly visualised (LAO 40ø, cranial 20ø). (C) This view is taken from LAO 0ø and cranial 40ø. Again only the left anterior descending artery is seen. (D) The right coronary artery continues in the posterior atrio-ventricular groove and terminates in the left atrial branch. The superdominant right coronary artery (LAO 0ø cranial 0ø). LAO : left anterior oblique.

She underwent elective coronary angiography with a standard right femoral approach. The left coronary angiogram showed only the left anterior descending coronary artery (LAD) arising from the left sinus of Valsalva and no LCX was seen with a left injection (Fig. 1a–c). The right coronary artery (RCA) originated normally from the right sinus of Valsalva. It had a normal course and continued beyond the crux through the left atrio-ventricular sulcus, running the full course of the LCX and terminating in the left atrial branch (Fig. 1d). Neither aortography nor pulmonary angiography showed a separate ostium for the LCX. The RCA supplied blood to the posterolateral and lateral walls of the left ventricle, so it was considered a super-dominant RCA. There was no stenosis in either the LAD or RCA.

**Fig. 2.**
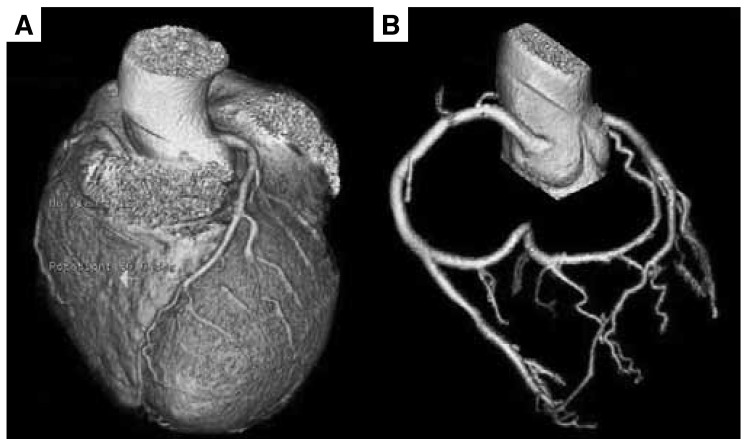
The circumflex artery is absent and only the left anterior descending artery is seen in the multi-detector computed tomography. (A) The left circumflex artery is absent in this view and the left anterior descending artery is clearly visualised. (B) A super-dominant right coronary artery is seen. The terminal portion of the right coronary artery unusually continues in the region of the left circumflex artery.

A stress thallium test was done which did not show ischaemia. An MDCT (Light Speed VCT; GE Healthcare, Milwaukee, Wisconsin, USA) was performed and the diagnosis wasconfirmed (Fig. 2).

## Discussion

Coronary artery anomalies are present at birth, but relatively few are symptomatic during childhood and later in life. Yamanaka et al. reported that only 20% of them are clinically significant.^1^ The LCX originating from the right sinus of Valsalva or as a proximal branch of the RCA is the second most common anomaly of the coronary arteries, with a reported incidence of 0.30 to 0.67%.^17^ On the other hand, absence of the LCX and a super-dominant RCA crossing the crux, ascending to the left atrio-ventricular groove and running the length of the LCX as a terminal extension of the RCA is an extremely rare entity, with a reported incidence of 0.003%.^1^

Because of different therapeutic options, a congenitally absent coronary artery must be distinguished from a totally occluded coronary artery that fills from another coronary artery via collaterals. Although an experienced operator can easily differentiate the two conditions, it is worth remembering the key issues. Firstly, coronary collaterals may be totally occluded, but not in a case of a congenitally absent coronary artery. Secondly, with total occlusion there are usually wall motion defects in the left ventricle, but not with a congenitally absent coronary artery. In addition, the largest vessel segment is usually the proximal segment of the totally occluded artery. However, in a congenitally absent coronary artery, the distal anastomotic segment seems to be the largest and the proximal segment may be relatively thinner. Therefore, in total occlusion, the proximal end of the artery ends sharply, whereas there is a tapered end of the vessel in congenital cases.^18^

The absence of the circumflex artery was first described by Baressi et al.^2^ in 1975 and to our knowledge, our case is the 17th in the literature. (The four patients in Yamanaka’s study and two patients in that of Komatsu are not included.)^1^-^16^,^19^ Except for one patient with myocardial infarction (but with no obvious narrowing of the coronary artery)3 and one patient with the co-existence of dilated cardiomyopathy,^4^ this abnormality is considered a benign congenital anomaly.

Itoi et al. found an abnormally low coronary flow reserve in the RCA of a 13-year-old patient with an absent LCX but no ischaemia on a treadmill test and no ischaemic area in the LCX region with a thallium test.6 Kursaklıoglu et al. has followed up on a patient with a Cx artery originating from the distal right coronary artery for 13 years and has reported a benign course.^8^ They also report that a thallium perfusion scan is superior to an exercise ECG for clinical decision making and follow up on these patients. In our patient, a normal stress thallium test and the absence of any plaque in the coronary arteries on angiography indicated the benign nature of this anomaly.

Although conventional cardiac catheterisation is a well-known test for the detection of coronary anomalies, the multi-detector row CT (MDCT) is emerging as an essential imaging tool for evaluating coronary arteries, as many of the congenital coronary anomalies are easily assessed with this modality.^20^ Because of its three-dimensional nature, MDCT is well suited to detect and define the anatomical course of coronary artery anomalies and their relationship to other cardiac and non-cardiac structures. The robust visualisation and classification of anomalous coronary arteries make CT angiography a first-choice imaging modality for the investigation of known or suspected coronary artery anomalies.^21^

The incidence of congenital absence of the left circumflex artery may be expected to be higher after wide usage of MDCT, however, Komatsu et al. reported only two cases (0.05%) of this type of anomaly in their 3 910 consecutive cases undergoing MDCT.^19^ This suggests the anomaly remains rare, even in the era of MDCT.

## Conclusion

The absence of the LCX with a super-dominant RCA running the full course of the LCX is a very rare anomaly and is not associated with adverse events. MDCT is a suitable non-invasive imaging modality for detecting congenital coronary anomalies.
